# Potential Use of Biotherapeutic Bacteria to Target Colorectal Cancer-Associated Taxa

**DOI:** 10.3390/ijms21030924

**Published:** 2020-01-30

**Authors:** Garreth W. Lawrence, Máire Begley, Paul D. Cotter, Caitriona M. Guinane

**Affiliations:** 1 Department of Biological Sciences, Cork Institute of Technology, Cork T12 P928, Ireland; Garreth.lawrence@mycit.ie (G.W.L.); Maire.begley@cit.ie (M.B.); 2 APC Microbiome Ireland, University College Cork, Cork T12 YN60, Ireland; paul.cotter@teagasc.ie; 3 Teagasc Food Research Centre, Moorepark, Fermoy, Cork P61 C996, Ireland

**Keywords:** colorectal cancer, microbiota, *Fusobacterium nucleatum*, probiotics, biotherapeutics

## Abstract

The role of the gut microbiome in human health and disease is the focus of much attention. It has been widely agreed upon that our gut bacteria play a role in host immunity, nutrient absorption, digestion, metabolism, and other key drivers of health. Furthermore, certain microbial signatures and specific taxa have also been associated with the development of diseases, such as obesity; inflammatory bowel disease; and, indeed, colorectal cancer (CRC), which is the focus of this review. By extension, such taxa represent potential therapeutic targets. In particular, the emerging human pathogen *Fusobacterium nucleatum* represents an important agent in CRC development and its control within the gastrointestinal tract is desirable. This paper reviews the principal bacterial pathogens that have been associated with CRC to date and discusses the in vitro and human studies that have shown the potential use of biotherapeutic strains as a means of targeting CRC-associated bacteria.

## 1. Introduction

Colorectal cancer (CRC) refers to cancer of the rectum or colon [1]. CRC accounts for 9.2% of all cancer diagnoses globally, corresponding to 1.096 million cases, and is the second leading cause of cancer-related deaths worldwide [2]. Colorectal tumorigenesis is attributed to genomic and/or epigenomic instability, resulting in the formation of neoplastic lesions [3,4]. More specifically, such instabilities can promote mutations that result in the inactivation of tumour suppressor genes and the activation of oncogenes, leading to colonic cell malignancies [5]. Risk factors for CRC include excessive alcohol consumption, smoking, and lack of exercise, and epidemiological studies have shown diets rich in red meat and animal fat may also promote CRC development [6] whereas a diet heavy in fruit and vegetables can have a protective effect [7]. Strong evidence has also been put forward for a role of specific human gut microbes in CRC development and progression [8,9,10,11].

Despite the advances in cancer therapeutics, including surgery and chemotherapy [12,13], in recent years, CRC remains one of the most aggressive and invasive malignancies [14]. Chemotherapy can damage healthy cells including hair follicle cells, mucous membranes of oral cavities, and the gastrointestinal tract, adversely affecting erythrocytes and leukocytes [15]. Surgical procedures such as colectomies may disrupt the resident gut microbiota, favouring the growth of harmful bacteria [16]. Therefore, nontoxic, noninvasive alternative therapeutics and, indeed, preventative approaches are highly desirable.

In this review, the microbial influence on the development of CRC is examined, with a focus on bacterial pathogens such as the emerging human pathogen *Fusobacterium nucleatum* that has been recently strongly associated with colon tumorigenesis. Bacterial strains that inhibit the CRC-associated pathogen *F. nucleatum* and that reduce CRC-associated taxa within the gut microbiota and, therefore, have the potential to contribute to CRC prevention by acting as biocontrol agents are also discussed. 

## 2. The Gut Microbiota of CRC Patients

The gut microbiome contains over 100 trillion microbes that play crucial roles in host health, and the maintenance of gut homeostasis is largely dependent on the symbiotic interactions between microbes and the digestive tract [17], while an imbalance in the gut microbiome can promote the secretion of bacterial toxins (virulence factors) and carcinogenic metabolites (hydrogen sulfide), decrease beneficial metabolites (butyrate), compromise the intestinal barrier function, cause immune dysregulation, and lead to cellular proliferation which can contribute to CRC [18].

Many studies have assessed the gut microbiome of CRC patients, revealing a number of patterns [19]. While there are variations in the microbial composition across different studies, detection methods, and sample types, several studies have consistently found increased abundances of the genus *Fusobacterium* in the gut microbiome of CRC patients [20,21,22,23,24,25,26]. While high *Fusobacterium* abundances are consistent, several other genera have also been found to be elevated in both tissue and stool samples of CRC, including *Peptostreptococcus* [20,21,22], *Streptococcus* [20,21,24], *Porphyromonas* [21,23], and *Selenomonas* [21,22]. In contrast, the butyrate (a short chain fatty acid (SCFA))-producing genus *Roseburia* is consistently reduced in CRC patients [20,21,22], while other butyrate-producing taxa were also underrepresented such as *Lachnospiraceae* [23] and *Ruminococcaceae* [25] (Table 1). Some representative studies are summarised here. For instance, Wang et al. [20] investigated the microbial composition of CRC patients in comparison to healthy controls using 16S rRNA amplicon (V3) sequencing of faecal samples. A clear alteration in microbial composition was observed, with an increase in the relative abundance of the genera *Fusobacterium*, *Enterococcus*, *Escherichia/Shigella*, *Klebsiella*, *Streptococcus*, and *Peptostreptococcus* and with a reduction in butyrate-producing bacteria, specifically *Roseburia*, in CRC patients. In another study, Gao et al. [21] observed similar trends using compositional 16S rDNA sequencing of cancerous tissue and found increased numbers of *Fusobacterium*, *Streptococcus*, *Peptostreptococcus*, *Porphyromonas*, and *Selenomonas*. Again, low levels of *Roseburia* were detected in comparison to healthy controls. Notably, there were significant differences in the relative abundances of *Fusobacterium* in malignant tissue compared to noncancerous mucosa, with 8.5% and 4.13%, respectively. Inconsistencies, however, are also evident between certain studies. For instance, Hibberd et al. [22] reported an enrichment of *Fusobacterium*, *Peptostreptococcus*, and *Selenomonas* and a depletion of *Roseburia*. However, the same study found lower abundances of *Streptococcus* in the tumour tissue of CRC patients, which was inconsistent with Gao’s findings. In another study, low abundances of *Lachnospiraceae* was significantly associated with CRC [23]. Indeed, Flemer et al. [27] postulated the protective effects of the butyrate-producing family *Lachnospiraceae* against CRC in an oral, faecal, and colonic mucosal metagenomic analysis. Butyrate, an intestinal microbial metabolite, demonstrates anti-CRC properties through several mechanisms including histone deacetylase (HDAC) inhibition and epigenetic modulation, which can restrict CRC cell proliferation and can induce apoptosis of CRC cells [28]. Butyrate plays an important role in host health through maintaining intestinal homeostasis, reinforcing the epithelial barrier, and inhibiting intestinal inflammation [29]. Thus, the depletion of butyrate-producing bacteria in the gut microbiota may increase the risk of CRC. 

The above subset of studies recognises that there are variations between studies. However, there are a number of consistent patterns that may provide biomarkers for diagnostic purposes and may highlight potential therapeutic targets. Reducing abundances of *Fusobacterium* and increasing butyrate-producing genera within the gut microbiota may serve as a preventative strategy for CRC. While high abundances of specific genera are associated with CRC, specific bacterial species have also been implicated in the disease. 

## 3. Specific Taxa Associated with CRC Development

Metagenomic analysis have revealed a significant increase in the presence of commensal bacteria in the gut microbiome of CRC patients including strains of *Escherichia coli*, *Bacteroides fragilis*, and in particular *F. nucleatum* [30,31,32]. Studies have also shown that specific species increase the risk of developing CRC. For example, a 2018 retrospective analysis of 13,096 patients found that CRC risk was increased in patients with bacteremia caused by *B. fragilis*, *Streptococcus gallolticus*, *F. nucleatum*, *Clostridium septicum*, or *Clostridium perfringens* [33]. Specific bacterial species and several mechanisms have been implicated in the development of CRC, supporting a causal role. For instance, pathogenic bacteria may promote CRC through the production of genotoxins and virulence factors, the upregulation of inflammatory pathways, and the initiation of oxidative stress, which ultimately lead to genetic instability and epithelial cell proliferation [17]. Therefore, controlling particular components of the gut may serve to reduce the risk of CRC development. The most extensively studied CRC-associated bacteria are *F. nucleatum*, *B. fragilis*, and *E. coli*. 

### 3.1. F. nucleatum 

*F. nucleatum* is a Gram-negative and strict anaerobe that predominantly colonises the oral cavity and is implicated in periodontal diseases such as gingivitis and periodontitis [34]. It is also associated with many other diseases and adverse health outcomes including inflammatory bowel disease (IBD), appendicitis, preterm births, stillbirths, atherosclerosis, rheumatoid arthritis, and Alzheimer’s [35,36,37,38,39,40,41]. Further to this, *F. nucleatum* has become an important human pathogen shown to be elevated in colonic tumours and localised with malignant colonic cells. An increased abundance of *Fusobacterium* species has been identified by transcriptomic and metagenomic profiling in colon cancer relative to healthy tissues [42,43,44,45]. Castellarin et al. [42] performed qPCR analysis on tumours of CRC patients and found an overrepresentation of *F. nucleatum* when compared to healthy tissue. High numbers of *F. nucleatum* have been found to correlate with the progression of colorectal carcinogenesis and molecular markers such as the CpG island methylator phenotype (CIMP) and colorectal tumours with evidence of microsatellite instability [46,47]. Park et al. [48] used 16S rRNA sequencing to compare the gut microbiota of patients with precursors to CRC and patients diagnosed with CRC for the first time. Mucosal biopsy samples of patients with tubular adenomas (TA), sessile serrated adenomas/polyps (SSA/P), and CRC revealed no differences in relative abundance of *Fusobacteria* between TA (4.3%) and SSA/P (1.9%) groups. However, a significant difference in the relative abundance of *Fusobacteria* was observed in the CRC group (33.8%) compared to TA and SSA/P groups. The *Fusobacterium*–CRC link was further strengthened, when Rezasoltani et al. [30] found an enrichment of *F. nucleatum* in adenomatous polyps compared with healthy matched controls. Ultimately, in 2018, a meta-analysis which included 122 studies associated high *F. nucleatum* numbers with the tumorigenesis pathway and the CpG island methylator phenotype [49]. Over time, evidence has continued to accumulate about the relationship between *F. nucleatum* and CRC going beyond a correlation, with the species now being viewed as a causative agent. 

The virulent potential of the species and its ability to disrupt natural cell activities are largely attributable to FadA, a characterised virulence factor [50]. Rubinstein et al. [51] further characterised this phenotype as being a promoter of CRC via regulation of E-cadherin (adhesion molecule) and the transcription factor β-catenin, leading to increased expression of oncogenic and inflammatory genes. Furthermore, Ma et al. [52] outlined the role of *F. nucleatum* infection in promoting carcinogenesis by interacting with E-cadherin in a normal colon epithelial cell line (NCM460) while enhancing proliferation and activating NF-κB signalling. Furthermore, *F. nucleatum* increased inflammation and tumour multiplicity in an Apc^Min/+^ mouse model of intestinal tumorigenesis [45]. *F. nucleatum* can promote inflammatory microenvironments by initiating the release of inflammatory cytokines [53]. Additionally, *F. nucleatum* can weaken host immune responses as antitumour immunity defences and natural killer (NK) cells are suppressed by *F. nucleatum*, ultimately inhibiting tumour killing [54]. Mima et al. [46] provided further evidence of immunosuppression when they correlated *F. nucleatum* and low-density CD3+ T-cells in colorectal carcinoma tissues. Further to this, *F. nucleatum* induced M2 polarization of antitumour macrophages, resulting in poor patient prognosis [55]. Causality was also demonstrated using RNA-seq technologies and functional assays to reveal that autophagy pathways were activated by *F. nucleatum* in CRC cells while apoptosis was inhibited. Interestingly, in the same study, *F. nucleatum* was shown to promote chemoresistance, which may explain why high abundances of *F. nucleatum* correlate with poor patient prognosis [56]. Thymocyte selection-associated high-mobility group box (TOX) is a highly conserved member of a family of DNA-binding proteins, which plays a role in cellular functions such as apoptosis, growth, DNA repair, and metastasis [57]. TOX expression was found to be reduced in *F. nucleatum*-positive malignant tissues of the colon compared with *F. nucleatum* negative tissues. The same study also supported recent findings associating *F. nucleatum* with low T cell densities, reporting low CD4^+^ T cell density in *F. nucleatum*-positive malignancies compared to *F. nucleatum*-negative controls [58]. 

As noted, the aforementioned findings provide an insight into the role of *F. nucleatum* in the activation and progression of tumorigenesis through specific cancer pathways. By extension, reducing *F. nucleatum* colonisation in the gut and colon may reduce the risk of CRC development and, once CRC is established, may slow the progression of the disease. However, other species are also implicated in CRC. 

### 3.2. B. fragilis

*B. fragilis* is a Gram-negative obligate anaerobe, ubiquitous in nature, and a predominant species in the gut microbiota [59]. While there are strains of *B. fragilis* that contribute to gut health and are being assessed as potential probiotics [60], a possible association between other *B. fragilis* and CRC has also been uncovered. For example, Purcell et al. [31] used qPCR analysis to show that elevated levels of Enterotoxigenic *B. fragilis* (ETBF) was associated with the early onset of colorectal carcinogenesis. *B. fragilis* is thought to promote colon carcinogenesis by stimulating cell proliferation and by inducing inflammation via the TH17-dependent pathway [61]. ETBF produces a 20-kDa metalloprotease virulence factor called *B. fragilis* toxin (BFT) [62]. The expression of BFT in mouse models induces persistent colitis, interferes with E-cadherin junctions, initiates β-catenin signalling, and activates IL-8 secretion in colonic epithelial cells (CECs) [63,64,65]. Sears et al. [66] reported that the NF-κB signalling pathway is initiated by BFT, leading to increased cell proliferation and initiation of inflammatory mediators resulting in mucosal inflammation and, ultimately, CRC. Recently, Chung et al. [67] used an intestinal tumorigenesis mouse model induced by *B. fragilis* in Apc^Min^ mice to demonstrate the pro-carcinogenic effects of BFT and its ability to activate NFκB and Stat3, facilitating tumorigenesis. The presence of the BFT gene has been considered a biomarker for CRC [68] and, therefore, also highlights a therapeutic target.

### 3.3. E. coli

*E. coli* is a natural habitant of the gut microbiota and, in most cases, a commensal organism. Indeed, some strains are known for their probiotic properties [69], such as the *E. coli* Nissle 1917 strain. However, several *E. coli* strains are known to be encode numerous virulence factors and many studies demonstrate the genotoxic activities of some *E. coli* that disrupt natural cellular events and, ultimately, induce apoptosis [70,71,72,73,74]. *E. coli* harbouring polyketide synthase (pks) genotoxic islands secrete colibactin, a bacterial toxin, which may cause DNA damage and promote genomic alterations and instability [75]. Numerous studies have identified elevated levels of *E. coli* in CRC patients. For example, Bonnet et al. [32] reported a significant increase in mucosa-associated or internalized *E. coli* in CRC tumours compared to noncancerous controls. Furthermore, various levels of cyclomodulin-producing *E. coli* were found across different stages of colon cancer, with stages III and IV higher in abundance than stage I. Metagenomic analysis of patients diagnosed with advanced carcinoma also supports the role of *E. coli* in CRC [76]. Arthur et al. [77] provided mechanistic insight by demonstrating that pks+ *E. coli* NC101 promotes colorectal carcinogenesis in azoxymethane (AOM)-treated IL-10^−/−^ mice. Furthermore, a recent study identified pks+ *E. coli* and ETBF to be predominantly colonising biofilms in the colonic mucosa of patients diagnosed with familial adenomatous polyposis compared to healthy controls. Interestingly, the same study showed that mice harbouring both colibactin-secreting *E. coli* and ETBF showed increased tumorigenesis compared to mice harbouring *E. coli* or ETBF individually [78]. These findings suggest that genotoxin-producing *E. coli* and *B. fragilis* could be a synergistic driver of colon cancer. 

### 3.4. Other Bacteria

While other bacteria have been associated with CRC, the evidence for causation is limited and further studies are needed. For example, the human pathogen *Salmonella enterica* Serovar Enteritidis (*S. enderitidis*) has also been associated with CRC in a limited number of studies. *S. enteritidis* expresses a secretion protein, AvrA, which initiates Stat3 and β-catenin signalling pathways, promoting cell proliferation and tumorigenesis [79]. An epidemiological study found increased risk of developing CRC after *S. enteritidis* infection relative to individuals who have not experienced infection by the pathogen [80]. Another example is *Enterococcus faecalis*, an emerging human pathogen of the GI tract [81], which was reported to be significantly more prevalent in patients with CRC compared to healthy control subjects [20,82]. However, its role in CRC is debated as some studies suggest protective properties; for example, heat-killed *E. faecalis* EC-12 was shown to decrease tumour size in the small intestine of a mouse model via downregulating β-catenin signalling [83]. This paradox likely reflects the considerable differences in the pathogenic potential of *E. faecalis* depending on the gene complement present.

**Figure 1:**
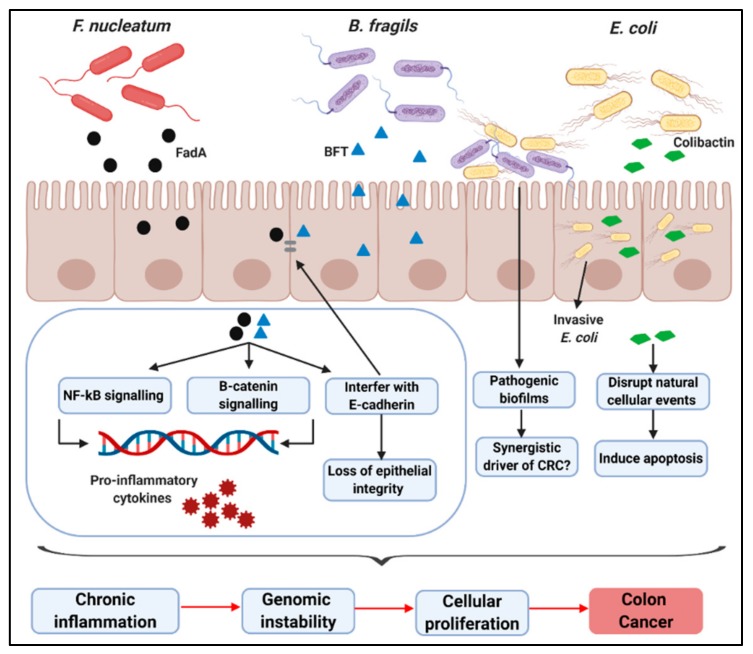
Overview of proposed mechanisms implicating principle CRC-associated bacterial pathogens in CRC development: Specific bacterial species may promote CRC through several mechanisms, such as the secretion of virulence factors by *F. nucleatum* (FadA) and *B. fragilis* (BFT), which activate NF-kB and β-catenin signaling pathways, leading to the development of pro-inflammatory cytokines which promotes pro-inflammatory microenvironments. Virulence factors may interfere with E-cadherin, a transmembrane protein essential for maintaining the epithelium cell layer, thus mediating the invasion of pathogenic bacteria. Cyclomodulin and genotoxin-producing *E. coli* (colibactin) may interfere with natural cellular events and may induce apoptosis. Biofilms predominantly colonized by genotoxin-producing *E. coli* and BFT-producing *B. fragilis* may be a synergistic driver of CRC. These events culminate in the development of chronic inflammation and genomic instability, which leads to epithelial cellular proliferation and, ultimately, CRC. This figure was created with BioRender.

In summary, the proposed mechanisms implicating bacterial pathogens in CRC are through bacterial toxin secretion, activation of inflammatory pathways, disruption of protection barriers, and interference of natural cellular events, resulting in chronic inflammation and genomic instability, which can lead to epithelial cellular proliferation and, ultimately, CRC; see Figure 1. 

## 4. Potential Use of Biotherapeutic Bacteria to Target Colorectal Cancer-Associated Taxa

The need for alternative therapies in this antibiotic resistance era is ever increasing. As the mechanisms through which specific bacterial species contribute to CRC are revealed, new strategies can be employed to reduce the growth of these species or, indeed, to eliminate them from the gut microbiota without harming beneficial bacteria in the process. Probiotics are now widely considered as a biotherapeutic and are defined as “live microorganisms that, when administered in adequate amounts, confer a health benefit on the host” [84]. Probiotic bacteria have potential as alternatives to antibiotics to control undesirable microbes through several mechanisms including antimicrobial (bacteriocin) production, competition for nutrients, and competitive exclusion of pathogens. Some antimicrobial-producing probiotics possess narrow spectrum activity against specific pathogenic bacteria and, thus, do not harm beneficial bacteria within the host microbiota. With regard to CRC, numerous clinical trials have shown the potential of probiotics as adjuvants, reducing the side effects associated with chemotherapy and surgeries in CRC-patients [85,86]. The delivery of live biotherapeutic cells for the treatment of GI diseases is well known and the immunomodulatory and anti-inflammatory properties of probiotics on colon cancer is well understood [87]; however, few studies exist that demonstrate the potential of probiotics to eradicate CRC-associated bacteria from the gut microbiome, consequently reducing the risk of disease development. 

## 5. Antimicrobial Activity of Biotherapeutic Strains against *Fusobacterium nucleatum*

The correlation between *F. nucleatum* and CRC has been extensively demonstrated, and the potential mechanisms underlying the promotion and progression of the disease have been elucidated. There is little doubt that targeting this organism in the GI tract and that the potential development of microbiota-targeted therapies against *F. nucleatum* are promising research avenues. As *F. nucleatum* was first identified as a pathogen associated with periodontal disease, numerous studies demonstrate the potential use of oral strains to inhibit many dental pathogens in vitro, including strains of *F. nucleatum* [88,89,90,91]. For example, bio-yogurt was shown to have antimicrobial activity against a range of periodontal pathogens, including the *F. nucleatum* strains tested. Furthermore, *Lactobacillus bulgaricus*, *Streptococcus thermophilus*, *Lactobacillus acidophilus*, and *Bifidobacterium* spp. isolated from bio-yogurt demonstrated antimicrobial activity against oral pathogens by competition assays. Notably, *Bifidobacterium* and *Streptococcus thermophilus* demonstrated inhibitory activity against *F. nucleatum* [90]. Another study assessed the antibacterial activity of a number of strains against a range of oral pathogens by inoculating antimicrobial-producing lactic acid bacteria (LAB) with periodontal pathogens in 96-well plates and by evaluating growth inhibition by optical density (OD) measurements at 600 nm after 24 h of anaerobic incubation. *Weissella cibaria* CMU and two probiotic species isolated from commercial probiotic products, *Lactobacillus salivarius* and *Lactobacillus reuteri*, showed antimicrobial activity against *F. nucleatum* with more than 95% growth inhibition compared with other probiotic strains. Interestingly, *W. cibaria* CMU demonstrated the highest co-aggregation rate with *F. nucleatum* with 81.2% as compared with other cariogenic and periodontopathic bacteria [91]. Co-aggregation, a beneficial property of probiotics, is a process of attachment which influences multi-species biofilm formation and is considered a beneficial property of probiotic and other biotherapeutic strains, which may result in barrier formation that prevents colonisation of pathogenic bacteria [92]. The antimicrobial activity of probiotics against CRC-associated *F. nucleatum* has also been assessed in vitro. Guzel-Seydim et al. [93] reported the anti-*F. nucleatum* potential of kefir (produced from natural Kefir grains), commercial Kefir, and yogurt (produced from natural starter cultures) against *F. nucleatum* by disc diffusion assays. Natural kefir demonstrated the largest inhibitory effect against *F. nucleatum*. Kefir produced from kefir grains is considered a functional food and a source of potential probiotics. Some kefirs and associated strains have shown specific health benefits [94]. The microbial composition of kefir grains is complex; however, the genus *Lactobacillus* and specifically the species *Lactobacillus kefiranofaciens* can dominate [95]. Inhibition of *F. nucleatum* due to probiotic supplementation is also evident in vivo. For example, a randomised control trial, which included an oral microbiota analysis, revealed a reduction in several oral species including *F. nucleatum* subsp. *vincentii* after administration of probiotic strains *Lactobacillus reuteri* DSM 17,938 and PTA 5289 daily for 12 weeks [96]. Considering, the oral microbiota’s influence on GI health and disease [97] and that bacteria associated with CRC are also found in the oral cavity, these findings suggest potential for oral lactobacilli to be active against CRC-associated *F. nucleatum*. While these observations are promising, whether the anti-*F. nucleatum* activity demonstrated by these strains will translate into the dynamic conditions of the GI tract is yet to be confirmed. Furthermore, the impact of these anti-*F.nucleatum* strains on the surrounding gut microbiota and host health must be evaluated. 

## 6. Probiotic Intervention Modulates the CRC-Associated Microbiome

A reduction in cancer-associated taxa due to the administration of probiotics has also been revealed through human clinical trials [22,98]. A prospective controlled trial randomised 22 CRC patients into a group receiving a mixture of *Bifidobacterium longum, Lactobacillus acidophilus*, and *Enterococcus faecalis* at a concentration greater or equal to 1.0 × 10^7^ CFU/g three times daily or into a control group receiving maltodextrin. Samples of mucosa were obtained from colonic tumour sites throughout surgery, and noncancerous mucosa samples were collected from various colonic areas. Amplification of the 16S rRNA V3 region and pyrosequencing revealed a significant reduction in *Peptostreptococcus* and *Comamonas* and, most notably, a 5-fold decrease in *Fusobacterium* numbers after administration of the probiotic mixture. Additionally, Proteobacteria and *Enterococcus* were more abundant in patients receiving probiotic treatment [98]. In another study, the colonic microbiota of CRC patients was assessed after probiotic intervention with subjects receiving oral tablets twice daily, containing a high concentration of *Bifidobacterium lactis* Bl-04 and *Lactobacillus acidophilus* NCFM, compared to healthy controls. Faecal samples were collected pre-intervention and, at the time of surgery and 16S rRNA gene sequencing, were used to evaluate the microbial composition. Analysis revealed strong differences between the microbial composition of the mucosa and tumour tissue of patients receiving the probiotic compared to patients receiving no probiotics and to noncancerous controls. The taxa most associated with CRC, *Fusobacterium*, and *Peptostreptococcus* were less represented in the faecal samples of patients that received the probiotic. Additionally, butyrate-producing taxa *Clostridiales* spp, *Faecalibacterium, Eubacterium*, *Roseburia*, and *Lachnopira* were elevated in different sample types obtained from CRC patients receiving probiotic supplementation [22]. Although these studies were not species specific, it warrants further investigation into the use of microbial cocktails to control *F. nucleatum* and other CRC-associated bacteria. Ultimately, these studies show that strains exhibiting in vitro activity against *F. nucleatum* may exist and that there is the possibility that CRC-associated taxa may be manipulated through probiotic interventional therapy. However, targeting specific species associated with CRC while leaving beneficial taxa unharmed is desirable. Therefore, the influence of candidate biotherapeutic bacteria on the overall gut microbiome must be elucidated. Additionally, for the use of biotherapeutics, such as probiotics to target specific bacteria associated with CRC, they must be able to survive passage through the harsh conditions of the GI tract and to remain functionally unaltered. They must be evaluated for resistance to oral cavity enzymes, stomach pH, bile salts, pancreatic juices, and intestinal mucus while demonstrate an ability to adhere to epithelial tissue. Further investigation of these and other strains with activity against CRC-associated taxa has considerable merit.

## 7. Concluding Remarks

**Figure 2:**
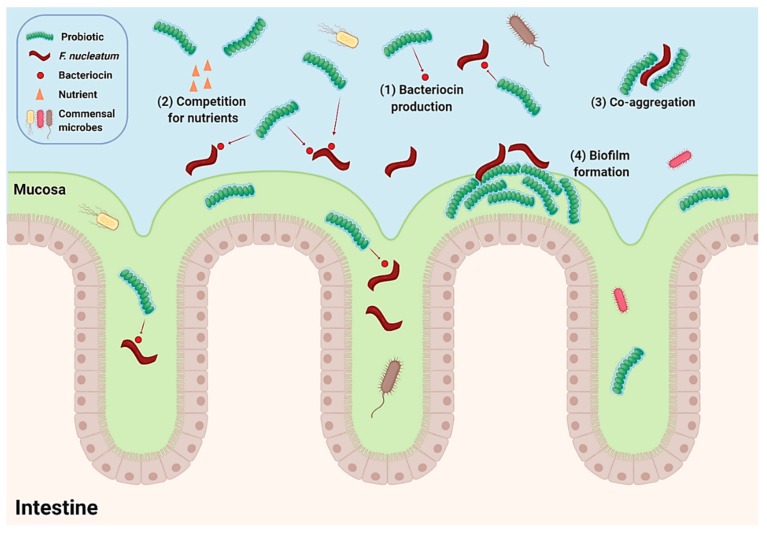
Proposed mechanism of action by which probiotic supplementation may decrease the risk of developing colon cancer by inhibiting *F. nucleatum* through (1) bacteriocin production, (2) competition for nutrients, (3) co-aggregation, and (4) competitive exclusion through biofilm formation. This figure was Created with BioRender.

Evidence associating bacterial species with CRC is rapidly increasing. Both metagenomic analysis and mechanistic studies continue to implicate bacterial pathogens in CRC development and disease progression. Thus, suppressing the growth of CRC-associated bacteria in the gut or colon may provide a strategy to reduce the overall risk of developing CRC. Further to this, eradicating the pathogens whilst leaving the surrounding gut microbiota unharmed is favourable. This may be achieved using narrow spectrum biotherapeutics, such as bacteriocin-producing probiotics. The above studies demonstrate the potential use of probiotic bacteria to target *F. nucleatum*, a bacterial pathogen associated with CRC. Although results obtained from in vitro studies do not necessarily translate into animal studies or human clinical trials, it offers potential for further research. The number of clinical trials evaluating the use of probiotics to manipulate specific bacterial species are limited, especially specific strains implicated in the CRC, but those that are available in the literature highlight the ability of probiotics to decrease abundances of *Fusobacterium*. Through the expression of antimicrobials and competitive exclusion, probiotics may serve as bio-controlling agents; see Figure 2. Their nontoxicity due to being natural residents of the gut microbiota further contributes to their safety and potential alternatives to antibiotics. Overall, the above studies demonstrate the use of probiotics to prevent the growth of *F. nucleatum* and to reduce CRC-associated taxa including *Fusobacterium*, which have been strongly associated with the development and progression of CRC and, therefore, showing promise in reducing the overall risk of CRC.Studies evaluating the use of probiotics to inhibit specific CRC-associated bacterial species have shown promising results for their use as a CRC-preventative strategy. Further research and clinical trials are needed to investigate the use of probiotics to target bacteria associated with CRC, positively impacting CRC outcomes.

## Figures and Tables

**Table 1:** Examples of microbial patterns observed in colorectal cancer (CRC) patients.

Sample Type		CRC Samples	Noncancer Controls	Detection Method	Probiotic/Antibiotic Exclusion Criteria	Overrepresented Taxa	Underrepresented Taxa	Reference
Tissue		65	65	16S rRNA gene (V4) pyrosequencing	No probiotic or antibiotic exposure within 4 weeks	*Fusobacterium*, *Streptococcus*, *Peptostreptococcus*, *Porphyromonas*, and *Selenomonas*	*Roseburia*	[21]
Tissue		31 ^b^	20	16S rRNA gene (V3) pyrosequencing	No antibiotic exposure within 2 months or probiotic exposure within 2 weeks	*Firmicutes*, *Fusobacteria*, *Lactococcus*, and *Fusobacterium*	*Pseudomonas* and *Escherichia/Shigella*	[26]
Tissue		65	65	16S rRNA gene (V1–V3) pyrosequencing	-	*Fusobacterium*, *Leptotrichia*, and *Campylobacter*	*Ruminococcus*, *Parabacteroides*, *Pseudoflavonifractor*, *Ruminococcaceae*, and *Holdemania*	[25]
Tissue and Mucosa		15	21	16S rRNA gene (V4) amplicon sequencing	Recent antibiotic exposure or regular use of probiotics	*Fusobacterium*, *Peptostreptococcus*, and *Selenomonas*	*Roseburia* and *Streptococcus*	[22]
Faecal	9 ^a^	49	Next Generation Sequence (NGS) analysis & terminal restriction fragment length polymorphism (T-RFLP)		Current use of antibiotics or regular use of probiotics	*Actinomyces*, *Atopobium*, *Fusobacterium*, *Haemophilus*, *Bacteroides fragilis*, *Clostridium nexile*, *Actinomyces odontolyticus* *Heamophilus parainfluenzae*, *Fusobacterium varium*, *Prevotella stercorea*, *Veillonella dispar*, and *Streptococcus gordonii*	*Slackia* and *Eubacterium coprostanoligens*	[24]
Faecal	46	56	16S rRNA gene (V3) pyrosequencing		No antibiotic exposure within 3 months	*Fusobacterium*, *Enterococcus*, *Escherichia/Shigella*, *Klebsiella*, *Streptococcus*, and *Peptostreptococcus*	*Roseburia*	[20]
Faecal	42	89	16S rRNA gene (V3–V4) pyrosequencing		-	*Fusobacterium* and *Porphyromonas*	*Clostridia* and *Lachnospiraceae*	[23]

^a^ Fifty samples from patients with colon adenomas were also obtained. ^b^ Samples were segregated into proximal and distal colon samples.
